# Interactions in Electrodeposited Poly-3,4-Ethylenedioxythiophene—Tungsten Oxide Composite Films Studied with Spectroelectrochemistry

**DOI:** 10.3390/polym13101630

**Published:** 2021-05-18

**Authors:** Aleksandra O. Efremova, Elena G. Tolstopjatova, Rudolf Holze, Veniamin V. Kondratiev

**Affiliations:** 1 Institute of Chemistry, Saint Petersburg State University, 7/9 Universitetskaya Nab., 199034 Saint Petersburg, Russia; alex-nize@rambler.ru (A.O.E.); e.tolstopyatova@spbu.ru (E.G.T.); rudolf.holze@chemie.tu-chemnitz.de (R.H.); 2 Institute of Chemistry, AG Elektrochemie, Chemnitz University of Technology, Straße der Nationen 62, 09111 Chemnitz, Germany; 3 State Key Laboratory of Materials-Oriented Chemical Engineering, School of Energy Science and Engineering, Nanjing Tech University, Nanjing 211816, China

**Keywords:** electronic absorption spectra, spectroelectrochemistry, poly-3,4-ethylenedioxythiophene film, tungsten oxide, composite film, propylene carbonate solutions

## Abstract

Cyclic voltammograms and optical absorption spectra of PEDOT/WO_3_ composite films were recorded in order to identify possible interactions and modes of improved performance of the composite as compared to the single materials. Changes in the shape of redox peaks related to the W(VI)/W(V) couple in the CVs of WO_3_ and the composite PEDOT/WO_3_ films indicate electrostatic interactions between the negatively charged tungsten oxide species and the positively charged conducting polymer. Smaller peak separation suggests a more reversible redox process due to the presence of the conducting polymer matrix, accelerating electron transfer between tungsten ions. Electronic absorption spectra of the materials were analyzed with respect to changes of the shapes of the spectra and characteristic band positions. There are no noticeable changes in the position of the electronic absorption bands of the main chromophores in the electronic spectra of the composite film. Obviously, the interactions accelerating the redox performance do not show up in the optical spectra. This suggests that the existing electrostatic interactions in the composite do not significantly change the opto-electronic properties of components of the composite but resulted in the redistribution of fractions of polaron and bipolaron forms in the polymer.

## 1. Introduction

In recent years growing attention has been paid to new hybrid materials combining organic and inorganic compounds. In particular, conducting polymers as a conductive matrix in combination with transition metal oxides have been widely investigated as novel functional materials for different applications such as electrochromic materials [1], energy storage materials [2,3,4] and photocatalysts [5].

Among the inorganic materials, tungsten oxide has been frequently studied as an electrochromic negative electrode [6,7,8] and energy storage material [8,9,10] with good chemical stability and strong adherence to the substrate.

Tungsten oxide WO_3_ can be obtained in different crystallographic structures, that are composed of three-dimensional networks of WO_6_ octahedra in corner-sharing or edge-sharing arrangements. This provides abundant channels or chains of interstitial sites for insertion of small ions [11], which are associated with the origin of electrochromism.

The kinetics of redox processes and the degree of reduction of tungsten oxides strongly depend on their structure and, in turn, on the synthesis procedures. In many investigated cases, the kinetics of the redox response of WO_3_ remain relatively sluggish due to slow diffusion in compact structures of tungsten oxides. Therefore, intrinsically conducting polymers have been employed recently as a matrix for incorporated WO_3_ particles to facilitate charge transport to the oxide.

In particular, the incorporation of WO_3_ nanostructures into polyaniline (PANI) [12,13,14,15,16,17], polypyrrole (PPY) [18] and poly(3,4-ethylenedioxythiophene) (PEDOT) [3,19,20,21] yielding composites has been reported; for an overview see [4]. The main achievements noticed with such combination is the acceleration of redox processes in the WO_3_ fraction due to faster charge transport across the polymer layer to the metal oxide and higher surface-to-volume ratio in the obtained composite structures further facilitating ion transport. The synthesis of nanostructured WO_3_/conducting polymer composites leads to synergistic enhancement of their electrochromic and pseudo-capacitive properties, combining the benefits from both components. For example, both components of PEDOT/WO_3_ composite (PEDOT and WO_3_) undergo blue coloration during the reduction process, thus ensuring the complementarity of the electrochromic responses and higher coloration efficiency.

An important question hardly addressed in most studies of composite materials aims at conceivable interactions between the constituents of the composite materials [4]: Are there any? Are they pronounced? What are their effects? It may also be of interest whether they will lead to new properties of the material. On the other hand, it is also possible that the composite simply inherits the properties of the original components (additive behavior), suggesting that the electronic structure and properties of the components is maintained in the composite.

In this report, we focus on a comparative study of cyclic voltammetry and electronic absorption spectra of single component films (PEDOT, WO_3_) and their composite material (PEDOT/WO_3_) at different electrode potentials in non-aqueous electrolyte solutions, in order to identify the contributions of redox processes and optoelectronic absorption and to conclude on the possible interaction between the components conceivably affecting their performance as supercapacitor electrode material. To our knowledge, such combined electrochemical and spectroelectrochemical investigations of PEDOT/WO_3_ composite films prepared by electrochemical deposition have not been reported.

However, an in-depth investigation of the electrochromic properties of film-coated electrodes and a full characterization of their electrooptical response was beyond the scope of our study. Instead, we used a spectroelectrochemical approach as a tool of independent evaluation of possible interactions between components of the formed composite and their impact on optical properties. Nevertheless, such full characterization, including further experiments and taking into account electrolyte solution effects as addressed elsewhere ) [22], are planned to be performed in the future, aiming at proper optimization of the composite.

## 2. Materials and Methods

3,4-ethylenedioxythiophene (EDOT, 98%) and lithium perchlorate (LiClO_4_, 99.9%) were purchased from Sigma-Aldrich (Darmstadt, Germany). Acetonitrile (HPLC grade, water content below 0.05%) was obtained from abcr GmbH (Karslruhe, Germany), propylenecarbonate (PC, 99%) from Alfa Aesar (Kandel, Germany). Sodium tungstate dihydrate (Na_2_WO_4_∙2H_2_O) and 18 M solution of H_2_SO_4_ were from Neva Reactive Co., Russia. All aqueous solutions were prepared using deionized water of resistivity > 18 MΩ·cm^−1^ (Merck Millipore, Darmstadt, Germany).

The electropolymerization and all electrochemical studies were conducted in a three-electrode cell at room temperature (20 ± 2 °C) using a PGSTAT302N potentiostat/galvanostat (Metrohm Autolab, Utrecht, The Netherlands) controlled by GPES software.

To produce optically transparent working electrodes (FTO-electrodes) SnO_2_/F-coated glass slides from Sigma-Aldrich (transmittance 82–84.5% (in the visible range), surface resistivity ~ 13 Ω cm^−2^) were cut to pieces with sizes of about 50 mm × 8 mm. Before film deposition, the FTO-electrodes were cleaned ultrasonically in ethanol for 20 min, washed with deionized water, and dried.

PEDOT was electropolymerized galvanostatically on FTO-electrodes at *j* = 1 mA·cm^−2^ from solutions of 0.05 M 3,4-ethylenedioxythiophene (EDOT) and 0.5 M of lithium perchlorate (LiClO_4_) in acetonitrile. To obtain relatively transparent films, the time of electrodeposition was limited to 100 s. Deposited amounts of both constituents were the same for the single constituent and the composite electrodes, in order to enable quantitative evaluation.

Tungsten oxide was electrodeposited either on pristine FTO-electrodes or on the pre-formed FTO/PEDOT film electrodes from a metastable acidic solution of isopolytungstate containing 0.005 M Na_2_WO_4_ and 0.5 M H_2_SO_4_ following a procedure described in [20,21], i.e., under potentiodynamic conditions within the potential range −0.3 to 0.7 V at a scan rate of 50 mV·s^−1^; the number of cycles was usually 60.

After film deposition, the WO_3_ and PEDOT/WO_3_ electrodes were carefully rinsed with PC, dried in air first at ambient temperature, then at an increased temperature (80 °C). The redox cycling of electrodes was performed in an aprotic solution 0.5 M LiClO_4_/PC in the potential range −0.6 to +0.8 V (vs. Ag/AgCl electrode) at scan rates 10–100 mV/s.

The spectroelectrochemical studies were performed in situ using a Shimadzu UV-1700 spectrophotometer (Japan) coupled to the PGSTAT302N potentiostat/galvanostat. The electronic absorption spectra were acquired at selected constant potentials of film electrodes in 0.5 M LiClO_4_/PC supporting electrolyte in the UV-vis-NIR range 300–1100 nm.

The spectroelectrochemical studies were performed using a custom-made three-electrode cell, comprising a 10 mm quartz cuvette (Hellma Müllheim, Germany) with a PTFE insert, supporting the vertically inserted FTO working electrode, a Pt foil counter electrode and an Ag/Ag^+^ reference electrode, the latter two arranged so that they did not block the light beam. For more convenient data presentation, the potential values of the Ag/Ag^+^ non-aqueous reference electrode were recalculated to the aqueous Ag/AgCl electrode.

The morphology of prepared composites was characterized by scanning electron microscopy (SEM, SUPRA 40VP Carl Zeiss, Oberkochen, Germany). EDX analysis was performed with an energy-dispersive X-ray spectrometer X-act (Oxford Instruments, Oxon, UK).

## 3. Results

### 3.1. Morphology

The morphology of tungsten oxide and the PEDOT/WO_3_ composite deposited on FTO and the localization of tungsten oxide deposits was studied with scanning electron microscopy and local EDX analysis.

**Figure 1:**
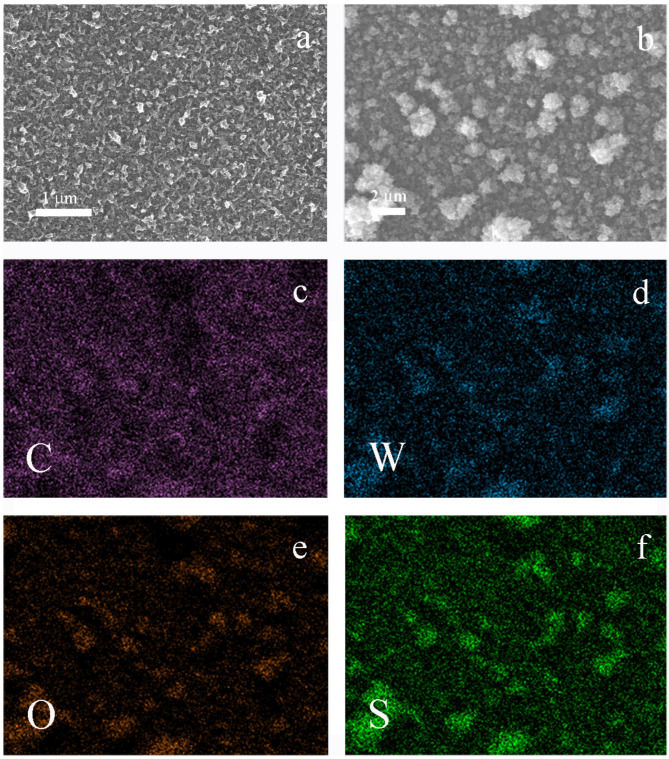
(**a**) SEM of PEDOT film; (**b**) SEM of PEDOT/WO_3_ composite film; (**c**–**f**) EDX element mapping of PEDOT/WO_3_ composite film as shown in Figure 1b.

With pristine PEDOT films, a rather typical globular structure of the polymer globules of about 50–200 nm sizes were observed (Figure 1a). During the electrodeposition of tungsten oxide, these globular structures were covered with a thin deposit of nanometer sized lamellar WO_3_ formations (100–500 nm), resulting in cauliflower-like agglomerates up to 2–3 μm size (Figure 1b).

EDX element distribution maps (C, W, O, S) across the surface of the scanned areas of the PEDOT/WO_3_ composite electrode were obtained (Figure 1c–f). The coincidence of the relative distribution density of sulfur from PEDOT and tungsten indicates the predominant deposition of tungsten oxide on dense globular supramolecular structures of the polymer.

### 3.2. Cyclic Voltammetry

**Figure 2:**
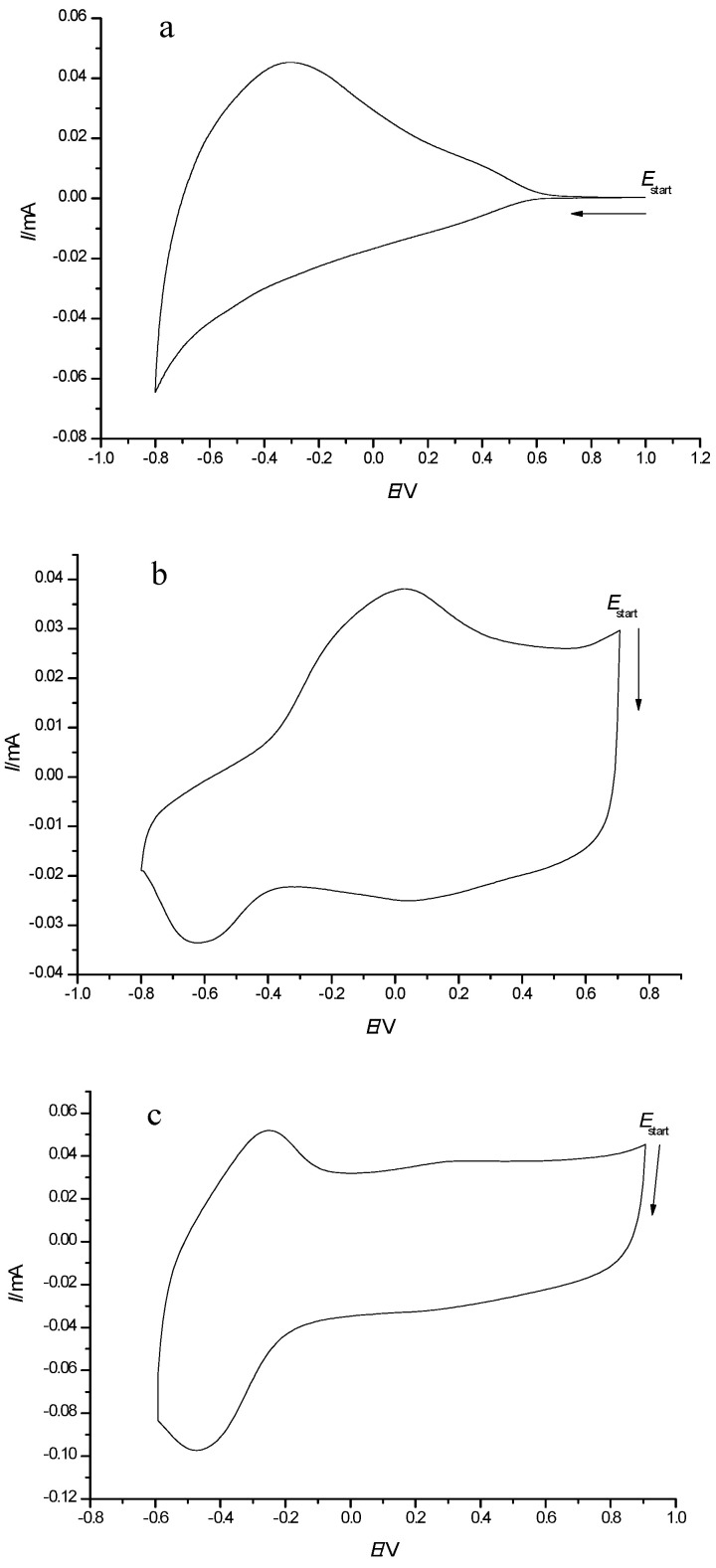
Cyclic voltammograms of: (**a**) FTO/WO_3_, (**b**) FTO/PEDOT, and (**c**) FTO/PEDOT/WO_3_ composite film electrodes in 0.5 M LiClO_4_/PC, *v* = 50 mV·s^−1^.

Typical CV responses of WO_3_, PEDOT and composite PEDOT/WO_3_ film electrodes in LiClO_4_/PC aprotic electrolyte solution are shown in Figure 2. The stability of PEDOT, WO_3_ and composite PEDOT/WO_3_ films was tested by long-term voltammetric cycling of film electrodes in the potential range from −0.1 to + 0.9 V. The peak currents were rather stable, showing a small gradual decrease with an increasing number of cycles. For example, continuous potential cycling of PEDOT and PEDOT/WO_3_ electrodes over 30 cycles lead to gradual decrease in the currents related to tungsten oxide less than 10%, whereas for WO_3_ electrodes, the stability was much better (decrease within 1–2%). The obtained result suggests a moderate stability of the composite electrodes. 

By comparison of CV shapes, it can be confirmed that both components, WO_3_ and PEDOT, are present in the PEDOT/WO_3_ composite film; however, in the composite film, their particular responses have changed already suggesting specific interactions.

The CV of the tungsten oxide film (Figure 2a) shows a rather broad redox response without a distinct cathodic peak and with a pronounced anodic peak at about −0.3 V. This type of CV response is characteristic for tungsten oxide deposited by different methods on FTO- or ITO-substrates. It is related to the reversible redox process W(VI)/W(V) in tungsten oxide, accompanied by the intercalation/deintercalation of Li^+^ ions into/out of the WO_3_ films with the formation of nonstoichiometric intercalated tungsten oxides according to:

WO_3_ + xLi^+^ + xe^−^ = Li_x_ [W(6+)]_1−x_[W(5+)]_x_O_3_ (1)

Figure 2b shows the characteristic CV of PEDOT in an organic electrolyte solution, with a broad anodic peak and two cathodic peaks.

The cyclic voltammogram of the PEDOT/WO_3_ composite film (Figure 2c) shows a pair of peaks (around *E*_0_ = −0.3 V) due to WO_3_ redox processes and a pseudo-capacitive response without any distinct peaks in the more positive potential range −0.1 to + 0.9 V, characteristic for PEDOT charging/discharging processes. As seen in Figure 2c, more pronounced and symmetric redox peaks of WO_3_ with smaller peak-to-peak separation are observed in the case of the PEDOT/WO_3_ composite film. The pronounced in the shape of CV response related to the W(VI)/W(V) redox couple indicates interactions between the constituents of the composite. The improved reversibility of the redox process of WO_3_ is due to the presence of the conducting polymer matrix, which accelerates electron transfer between tungsten ions. Apparently, electrostatic interactions between the negatively charged tungsten oxide species and the positively charged oxidized fragments of conducting polymer take place.

Thus, it can be stated that currents are not simply additive in the overall electrochemical response of the composite film.

### 3.3. Spectroelectrochemistry

The spectra of PEDOT/WO_3_ composite films, as well as their components (PEDOT and tungsten oxide films) were recorded in solutions of 0.5 M LiClO_4_ in propylene carbonate in the range 300–1100 nm at fixed electrode potentials in the range from −0.8 V to +1.0 V. All spectra were collected at stationary conditions (after the registered current remained almost unchanged).

**Figure 3:**
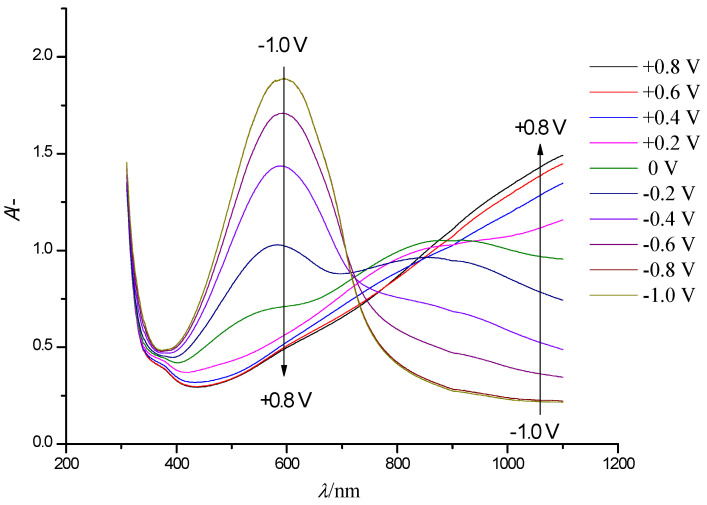
Electronic absorption spectra of a PEDOT film on an FTO electrode with a controlled change in the electrode potential in the range of −1.0 V to + 0.8 V (step 0.2 V, arrows show the direction of the absorption band change with increasing potential) in solution of 0.5 M LiClO_4_/PC.

Figure 3 shows a series of steady-state electronic absorption spectra of the PEDOT film. The arrows indicate the direction of the optical density variations in the case of gradual film oxidation (shift to positive potentials).

In the neutral state of PEDOT (E = −0.8 V), the main absorption band at 600 nm, related to π-π* interband electronic transitions in neutral film fragments [23,24] is dominant. During the oxidation of PEDOT, the absorbance at this wavelength decreases, and at potentials higher than +0.4 V it completely disappears. Instead, a new band with a maximum at 840 nm appears, which is assigned to a similar electronic transition in the first oxidation state of the polymer units–polaron species. When approaching the highest potential, the absorption growth at 840 nm slows down, and an edge of a new absorption band appears in the near IR NIR spectral range with a maximum located beyond the registered wavelength range (approx. *λ*_max_ > 1100 nm). It is noteworthy that this type of spectral evolution is typical for several thiophene derivatives at various degrees of oxidation [25,26,27,28,29], this suggesting that similar processes occur in the polymers even with chemically different substituents. Two isosbestic points are observed in the set of spectra: the first isosbestic point near *λ* = 720 nm occurs in the potential range from −0.8 to 0.2 V, while the further positive potential shift (E > 0.3 V) leads to the appearance of the second, poorly expressed isosbestic point (isosbestic range) around *λ*_max_ = 860 nm (Figure 3). The presence of isosbestic points is typical for mixtures of absorbing components/species with a constant overall concentration.

The presented spectroelectrochemical data suggest the presence of three types of absorbing species in the PEDOT film. This is in agreement with the well-known polaron-bipolaron model of redox processes in ICPs [30,31,32]. The oxidation of neutral PEDOT fragments (*λ*_max_ = 600 nm) leads to consecutive formation of oxidized species: polarons (*λ*_max_ = 860 nm) and consecutively bipolarons (*λ*_max_ > 1100 nm).

**Figure 4:**
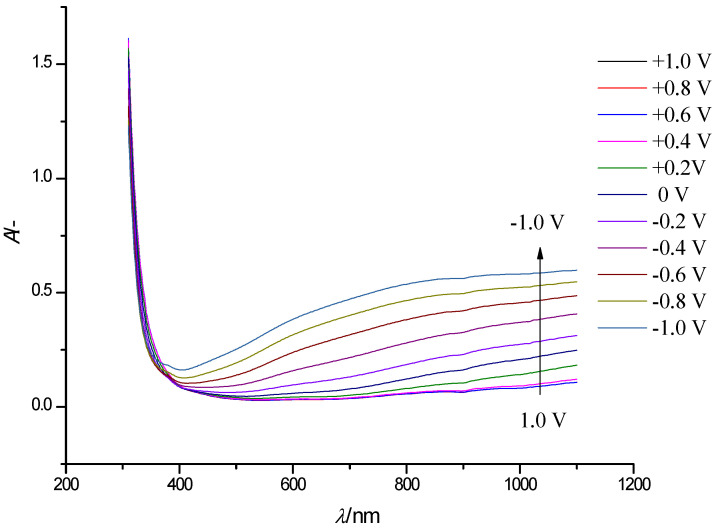
Electronic absorption spectra of a WO_3_ film on an FTO electrode at electrode potentials in the range −1.0 to +1.0 V (step width 0.2 V, arrows show the direction of the absorption band change with decreasing potential) in solution of 0.5 M LiClO_4_/PC.

Figure 4 shows a similar series of steady-state electronic absorption spectra of a WO_3_ film. In the spectra recorded at potentials ranging from 1 V to −0.8 V, we can see the appearance of a broad absorption band in the vis-NIR range of the spectrum (850–1100 nm), which grows with a negative shift of electrode potential.

Following gradual WO_3_ reduction, at potentials lower than +0.4 V, an additional broad absorption band was observed in the range of wavelengths 500–800 nm. Because of the considerable width of both absorption bands, a clear maximum of the second band can hardly be located; this prevents separation of the bands. Maximum absorption of WO_3_ in the visible and NIR range, and blue coloration was achieved at the most negative electrode potential −1.0 V (Figure 4). This is associated with the intercalation of the Li^+^-ions and electron flow into the WO_3_ film. The intense blue color is caused by intervalence charge transfer between adjacent W^5+^ and W^6+^ sites in tungsten oxide [33,34].

**Figure 5:**
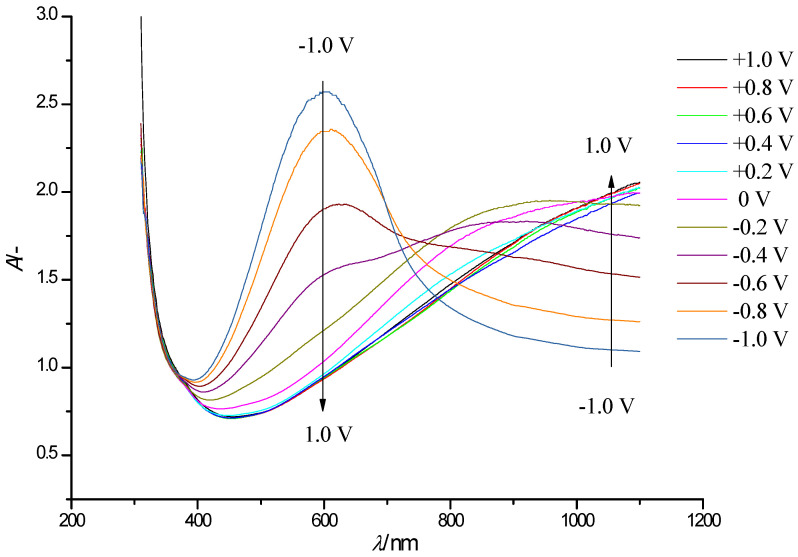
Electronic absorption spectra of a PEDOT/WO_3_ film on an FTO electrode at electrode potentials in the range −1.0 to + 1.0 V (step width 0.2 V, arrows show the direction of the absorption band change with increasing potential) in solution of 0.5 M LiClO_4_/PC.

There are three major absorption ranges in the spectra of PEDOT/WO_3_ composite film (see Figure 5): Range I: 400–720 nm, range II: 720–970 nm and range III: 970–1100 nm. Detailed analysis and comparison of absorption spectra of a PEDOT film and a PEDOT/WO_3_ composite film reveals the main features and nature of spectral changes.

In the spectra of the composite, in range I, the absorption of the PEDOT film dominates with an additional contribution from tungsten oxide to the overall absorption at potentials from 0.2 to −0.5 V. Range II (720–970 nm) undergoes the strongest change in the shape of the overall spectrum of the composite film compared to the plain PEDOT polymer film, associated with a noticeable absorption of tungsten oxide in addition to the contribution of PEDOT both in the visible part of the spectrum (at *λ* = 500–800 nm, with a maximum around *λ* = 600 nm), and in the NIR range. At negative potentials, both PEDOT and WO_3_ components absorb in the visible range, synergistically providing blue color of the PEDOT/WO_3_ film. This observation is consistent with the data on the enhancement of electrochromic properties due to complementary change of both constituents in the composite film in the color (blue) at negative potential values available in the literature [35].

There are also three characteristic bands in the in situ absorption spectra of the composite, showing intensity changes with the applied electrode potential (Figure 5). These bands dominate at λ_max_ about 600 nm, *λ*_max_ about 900 nm and *λ*_max_ > 1100 nm. At the first glance, there are no obvious changes of the wavelengths of the maxima of observed bands in the PEDOT/WO_3_ nanocomposite in comparison to the pristine PEDOT polymer film at relatively low electrode potentials. At negative potentials both, the absorption band of PEDOT and bands of WO_3_, contribute, but due to the flat bands of WO_3_, band shapes and peak locations of maxima from PEDOT are preserved.

This suggests that the inclusion of WO_3_ into the polymer matrix did not significantly change its electronic structure at a moderate level of polymer oxidation, and the electrostatic interactions noticed in CVs accelerating the kinetics of redox performance (reversibility of W(VI)/W(V) redox couple) do not show up in the optical spectra.

**Figure 6:**
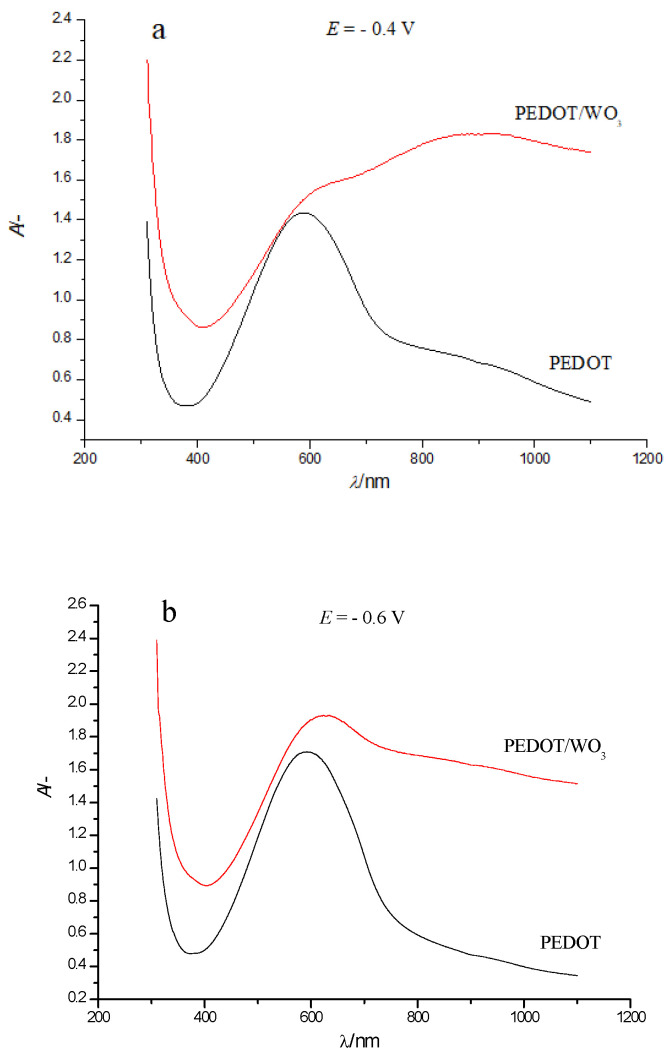
Comparison of electronic absorption spectra of PEDOT and PEDOT/WO_3_ films on an FTO electrode in solution of 0.5 M LiClO_4_/PC at selected electrode potentials: (**a**) −0.4 V, (**b**) −0.6 V.

The comparison of electronic absorption spectra of PEDOT and PEDOT/WO_3_ films at two selected potentials (Figure 6) clearly shows the differences between the spectra of PEDOT and PEDOT/WO_3_.

When comparing Figure 3 and Figure 5 more closely, we can also see that at the transition from neutral to oxidized state there is no expressed isosbestic point, like the one observed in the case of PEDOT only. In addition, the strongest changes in the shape of the spectrum of PEDOT/WO_3_ composite compared to the pristine PEDOT film are associated with a noticeable absorption of tungsten oxide both in the visible and NIR parts of the spectrum. In particular, at high positive potentials, where contributions from WO_3_ absorption is quite low (see Figure 4), the absorption in the NIR-range (*λ* ~ 1090 nm) from bipolaronic species of PEDOT in PEDOT/WO_3_ composite is much lower compared to pristine PEDOT film.

The reason for this decrease in absorption (so-called quenching) of bipolaronic fragments of PEDOT in the composite is probably the redistribution of fractions of polaron and bipolaron forms in the polymer, which resulted in the change of intensities of corresponding bands. Probably, at high positive electrode potentials of the composite film, the electrostatic interaction between the anionic form of tungsten oxide and the positively charged polarons leads to the stabilization of the polaronic species and a decrease in the fraction of bipolaronic forms in the polymer, which in turn results in lower intensity of the corresponding absorption band of the PEDOT/WO_3_ composite.

## 4. Conclusions

As noted above, the CVs and electronic absorption spectra of PEDOT/WO_3_ composite film are not the simple sum of individual components PEDOT and WO_3_ electrodeposited on FTO electrodes, as might have been expected for the case of a simple additive mixture. The shape of CV peaks attributed to the WO_3_ component in of PEDOT/WO_3_ composite film is significantly different from that observed with simple electrodeposited WO_3_. This is most likely caused by different mechanisms of electrodeposition of tungsten oxide on an FTO electrode and a PEDOT-covered FTO electrode. The difference was clearly observed before in a systematic investigation of electrodeposition conditions and their effects on the electrochemical response of tungsten oxide-covered FTO electrode and reported recently [36]. As shown, the potentiodynamic method of electrodeposition may result in trapping of polyoxotungstate anion species into the polymer during p-doping of the polymer and may thus facilitate preliminary formation of precursors of tungsten oxide nanostructures. The resulting precipitate of WO_3_ displays more symmetrical redox peaks with smaller peak-to-peak potential separation, this may be enhanced by the conductive matrix. These conditions and arguments do not apply to the case of electrodeposition on an FTO-surface from the same electrolyte solution.

Detailed analysis of electronic absorption spectra of PEDOT, WO_3_ films and PEDOT/WO_3_ composite films also suggests a situation more complex than simple additivity of the spectral components of the composite constituents. Although we do not see pronounced shifts of main electronic bands, which would indicate specific chemical interactions between the components, we nevertheless observe a redistribution of the fractions of different oxidized forms (polarons and bipolarons) of PEDOT. We propose that electrostatic interactions between positively charged polymer fragments and negatively charged polymer oxoanions of tungsten oxide lead to a stabilization of polaron species of the polymer and to a decrease in the degree of charge delocalization in the composite film. Therefore, at high positive electrode potentials of the composite film, the redistribution of fractions of polaron and bipolaron forms in the polymer was observed, which resulted in the change of intensities of corresponding bands. These observations shed light on performance improvements of these functional composite materials for both supercapacitor and optoelectronic applications.
